# Supplementary material for: Utility of Gene Panels for the Diagnosis of Inborn Errors of Metabolism in a Metabolic Reference Center
Source: Genes (Basel). 2021 Aug 19;12(8):1262. doi: 10.3390/genes12081262 (PMC8391361; doi:10.3390/genes12081262)
Supplement: Supplementary file 1 [file genes-12-01262-s001.zip › genes-1321555-supplementary.pdf]

Supplementary Table S1 - Clinical data and genetic findings for each patient for which a positive diagnosis was established using the INT MET panel.

Cases	Age	Sex	HPO	Gene	Variants	Phenomizer	Disorder<br>MIM	Changes in medication and/or diet*	Parents/Inheritance
1	2 m	M	Focal myoclonic seizures<br>EEG with burst suppression<br>Lethargy<br>Nonketotic hyperglycinemia<br>Intellectual disability	*AMT*	c.2T>C/<br>c.878-1G>A	p=0.0040	Glycine encephalopathy<br>#605899	Yes	Carriers<br>Autosomal recessive
2	9 y	M	Cerebral palsy<br>Epileptic encephalopathy<br>Epileptic spasms	*PCCA*	c.469-4A>G/<br>c.929C>G	p=0.4157	Propionic acidemia<br>#606054	Yes	Carriers<br>Autosomal recessive
3	-	M	Focal myoclonic seizures<br>EEG with burst suppression<br>Lethargy<br>Nonketotic hyperglycinemia	*GLDC*	c.2714T>G	p=0.0221	Glycine encephalopathy<br>#605899	Yes	*De novo*<br>Autosomal dominant
4	3 m	F	Seizures<br>Xanthine nephrolithiasis<br>Encephalopathy<br>Microcephalia<br>Hypouricemia<br>Growth delay	*MOCS2*	c.244A>T/<br>c.244A>T	p=0.0481	Molybdenum cofactor deficiency B<br>#252160	Yes	Carriers<br>Autosomal recessive
5	7 m	F	Anemia<br>Leukopenia<br>Thrombocytopenia<br>Gingivitis<br>Abnormality of homocysteine metabolism<br>Sepsis	*TCN2*	g.(?_31018929)_(31019095_?)del/<br>g.(?_31018929)_(31019095_?)del	p=0.0326	Transcobalamin II deficiency<br>#275350	Yes	Carriers<br>Autosomal recessive
6	1 y	F	Hyperphenylalaninemia<br>Abnormality tyrosine metabolism	*PAH*	c.842C>T/<br>c.490A>G	p=0.0018	Hyperphenylalaninemia<br>#261600	No	Carriers<br>Autosomal recessive
7	3 y	M	Hyperphenylalaninemia<br>Abnormality tyrosine metabolism	*PAH*	c.782G>A/<br>c.136G>A	p=0.0018	Hyperphenylalaninemia<br>#261600	No	Carriers<br>Autosomal recessive
8	7 m	M	Hypocalcemia<br>Vitamin D deficiency<br>Organic aciduria	*MCCC2*	c.1015G>A/<br>c.1423G>A	p=0.0458	3-Methylcrotonyl-CoA carboxylase 2 deficiency<br>#210210	Yes	Carriers<br>Autosomal recessive

			Elevated plasma acylcarnitine<br>Muscular hypotonia						
9	9 y	M	Hematuria<br>Hypercalciuria<br>Nephrolithiasis<br>Hyperuricemia	*HPRT1*	c.424A>G	p=0.0018	HRPT-related hyperuricemia<br>#300323	Yes	-<br>X-linked recessive
10	1 m	M	Abnormality of fatty-acid metabolism<br>Glutaric acidemia	*ETFDH*	c.1601C>T/<br>c.1601C>T	p=0.0481	Glutaric acidemia IIC<br>#231680	Yes	Carriers<br>Autosomal recessive
11	1 y	M	Hyperuricemia<br>Episodic ataxia<br>Xanthinuria	*MOCOS*	c.2356del/<br>c.512C>T	p=0.6593	Xanthinuria, type II<br>#603592	Yes	Carriers<br>Autosomal recessive
12	1 m	M	Hyperphenylalaninemia<br>Abnormality tyrosine metabolism	*PAH*	c.782G>A/<br>c.1066-11G>A	p=0.0018	Hyperphenylalaninemia<br>#261600	No	Carriers<br>Autosomal recessive
13	23 d	M	Hyperphenylalaninemia<br>Abnormality tyrosine metabolism	*PAH*	c.1162G>A/<br>c.728G>A	p=0.0018	Hyperphenylalaninemia<br>#261600	No	Carriers<br>Autosomal recessive
14	3 y	F	Hyperphenylalaninemia	*PAH*	c.898G>T/<br>c.1066-11G>A	p=0.3342	Hyperphenylalaninemia<br>#261600	No	Carriers<br>Autosomal recessive
15	13 d	F	Hyperphenylalaninemia<br>Abnormality tyrosine metabolism	*PAH*	c.1169A>G/<br>c.116_118delTCT	p=0.0018	Hyperphenylalaninemia<br>#261600	No	Carriers<br>Autosomal recessive
16	6 y	M	Hyperphenylalaninemia<br>Abnormality tyrosine metabolism	*PAH*	c.261C>A/<br>c.1315+1G>A	p=0.0018	Hyperphenylalaninemia<br>#261600	No	Carriers<br>Autosomal recessive
17	15 y	M	Myoclonus<br>Action tremor	*ATP7A*	c.2068G>C	p=0.2510	Menkes disease<br>#309400	Yes	NA<br>X-linked recessive
18	2 m	M	Methylmalonic acidemia	*ACSF3*	c.1672C>T/<br>c.1075G>A	p=0.2151	Combined malonic and methylmalonic aciduria<br>#614265	Yes	Carriers<br>NA
19	14 y	M	Encephalopathy<br>Hyperammonemia<br>Hyperbilirubinemia	*OTC*	c.903A>C	p=0.5477	Hyperammonemia due to ornithine transcarbamylase deficiency<br>#311250	Yes	Mother carrier, brother and maternal uncle affected<br>Autosomal recessive
20	18 y	M	Hepatic steatosis	*AMACR*	c.43delG/<br>c.43delG	p=0.7096	Congenital bile acid synthesis defect type 4<br>#214950	Yes	Carriers<br>Autosomal recessive

21	8 y	F	Hepatic steatosis<br>Hepatomegaly<br>Increased hepatic glycogen content	*PHKB*	c.2581A>G/<br>c.1459-9G>T	p=0.0036	Phosphorylase kinase deficiency of liver and muscle<br>#261750	Yes	Carriers<br>Autosomal recessive

** Changes in medication and/or diet after genetic diagnosis. d:days; F: female; m: month; M: male; NA: not available; y:years*

Supplementary Table S2 - Clinical data and genetic findings for each patient for which a positive diagnosis was established using the HYPO/HYPER panel.

Cases	Age	Sex	HPO	Gene	Variants	Phenomizer	Disorder MIM	Changes in Medication or Diet*	Parents/Inheritance
22	1 y	F	Hypoketotic hypoglycemia<br>Abnormality of carnitine metabolism	*SLC22A5*	c.34G>A/<br>c.1463G>A	p=0.0534	Carnitine deficiency, systemic primary<br>#212140	Yes	Carriers<br>Autosomal recessive
23	-	M	Hepatomegaly<br>Hypoglycemia<br>Motor delay<br>Growth delay	*PHKA2*	c.1245+1G>T	p=0.0160	Glycogen storage disease, type IXa<br>#306000	Yes	Mother<br>X-linked
24	-	M	Hypoglycemia<br>Vertigo<br>Polyphagia<br>MODY<br>Increased HbA1c levels	*CEL*	c.1810G>C/<br>c.1975G>C	p=0.0224	Maturity-onset diabetes of the young, type VIII<br>#609812	Yes	Carriers<br>Autosomal dominant
25	1 y	M	Ketotic hypoglycemia<br>Hepatomegaly<br>Increased hepatic transaminases<br>Myopathy	*AGL*	c.100delC/<br>c.100delC	p=0.0718	Glycogen storage disease III<br>#232400	Yes	Carriers<br>Autosomal recessive
26	6 y	M	Hepatomegaly<br>Hypoglycemia<br>Hypercholesterolemia<br>Elevated hepatic transaminases<br>Short stature	*PHKA2*	c.3547_3548ins<br>GGTGCCAT<br>GGACACCC<br>TGGAGA	p=0.0174	Glycogen storage disease, type IXa<br>#306000	Yes	-<br>X-linked
27	10 y	M	Hypergalactosemia<br>Galactosuria<br>Hypoglycemia	*GALT*	c.443G>A/<br>c.443G>A	p=0.0006	Galactosemia<br>#230400	No	Carriers<br>Autosomal recessive

28	1 m	M	Hypoketotic hypoglycemia<br>Hyperammonemia<br>Lethargy	*CPS1*	c.1201G>C/<br>c.2810T>A	p=0.0320	Carbamoyl phosphate synthetase I deficiency<br>#237300	Yes	Carriers<br>Autosomal recessive
29	-	M	Intrahepatic cholestasis<br>Elevated alkaline phosphatase<br>Hypercalcemia<br>Hyperphosphatemia<br>Vitamin D deficiency<br>Elevated hepatic transaminases<br>Hypoalphalipoproteinemia	*PHKA2*	c.3614C>T	p=0.0003	Glycogen storage disease, type IXa<br>#306000	Yes	-<br>X-linked
30	5 y	M	Hepatomegaly<br>Hypoglycemia<br>Fasting hypoglycemia	*PHKA2*	c.2746C>T	p=0.055	Glycogen storage disease, type IXa<br>#306000	Yes	-<br>X-linked
31	17 y	F	Abdominal pain<br>Macrovesicular hepatic steatosis	*ALDOB*	c.448G>C/<br>c.448G>C	p=0.005	Fructose intolerance, hereditary<br>#229600	Yes	Carriers<br>Autosomal recessive
32	8 m	F	Hyperinsulinemia<br>Abdominal distention<br>Fasting hypoglycemia<br>Intrauterine growth retardation	*INSR*	c.2621C>T/<br>c.3712C>T	p=0.0028	Hyperinsulinemic hypoglycemia, familial, 5<br>#609968	Yes	De novo/Mother<br>Autosomal recessive

* *Changes in medication and/or diet after genetic diagnosis .d:days; F: female; m: month; M: male; y:years*

Supplementary Table S3 - Clinical data and genetic findings for each patient for which a positive diagnosis was established using the MITO panel.

Cases	Age	Sex	HPO	Gene	Variants	Phenomizer	Disorder MIM	Changes in Medication or Diet*	Parents/Inheritance
33	3 y	F	Ventriculomegaly<br>Epileptic spasms<br>Hypoplasia of the corpus callosum<br>Severe expressive language delay<br>Slowly progressive spastic quadriparesis	*GFM1*	c.2011C>T/<br>c.1401delA	p=0.0302	Combined oxidative phosphorylation deficiency<br>#609060	No	Carriers<br>Autosomal recessive
34	9 y	M	Intrauterine growth retardation<br>Delayed CNS myelination<br>Athetosis<br>Lactic acidosis	*FOXRED1*	c.920G>A/<br>c.733+1G>A	p=0.1400	Mitochondrial complex I deficiency<br>#618241	No	Carriers<br>Autosomal recessive
35	18 y	M	Decreased activity of mitochondrial complex I and IV<br>Left ventricular hypertrophy<br>Abnormality of coordination<br>Dysdiadochokinesia<br>Specific learning disability	*TSFM*	c.719G>C/<br>c.719G>C	p=0.0032	Combined oxidative phosphorylation deficiency<br>#610505	No	Carriers<br>Autosomal recessive
36	-	F	Epileptic encephalopathy<br>Febrile seizures<br>Status epilepticus<br>Disinhibition<br>Global developmental delay	*GFM2*	c.1925A>C/<br>c.1922T>A	p=0.2282	Combined oxidative phosphorylation deficiency<br>#618397	No	Carriers<br>Autosomal recessive
37	6 y	F	Abnormal mitochondria in muscle tissue<br>Agenesis of corpus callosum<br>Cerebral atrophy<br>Increased CSF lactate<br>Global developmental delay<br>Seizures	*ADCK4*	c.418G>A	p=0.9010	Nephrotic syndrome, type 9<br>#615573	Yes	De novo<br>Autosomal dominant
38	6 y	F	Hypomimic face<br>Feeding difficulties<br>Bilateral ptosis<br>Optic atrophy<br>Muscular hypotonia<br>Areflexia	*DNM1L*	c.1337G>T	p=0.0288	Encephalopathy, lethal, due to defective mitochondrial peroxisomal fission 1<br>#614388	No	De novo<br>Autosomal dominant

39	24 h	M	Hypertrophic cardiomyopathy<br>Muscle weakness<br>Congenital cataract<br>Increased serum lactate	*AGK*	c.518+1G>A/<br>c.518+1G>A	p=0.0160	Sengers syndrome<br>#212350	No	Carriers<br>Autosomal recessive
40	-	M	Decreased activities of mitochondrial complexes I and III<br>Neonatal hypotonia<br>Global development delay<br>Seizures	*EARS2*	c.319C>T/<br>c.322C>T	p=0.0023	Combined oxidative phosphorylation deficiency 12<br>#614924	No	Carriers<br>Autosomal recessive
41	-	F	Hypertrophic cardiomyopathy<br>Hypotonia<br>Lactic acidosis	*OPA1*	c.1710T>G	-	Optic atrophy<br>#165500<br><br>Optic atrophy plus syndrome<br>#125250	No	De novo<br>Autosomal dominant
42	6 y	M	Muscular hypotonia<br>Myalgia<br>Ataxia<br>Head tremor<br>Limb tremor<br>Ptosis<br>Failure to thrive	*PDHA1*	c.787C>G	p=0.0366	Pyruvate dehydrogenase E1-alpha deficiency<br>#312170	Yes	De novo<br>X-linked dominant
43	8 m	F	Lactic acidosis<br>Generalized hypotonia<br>Global developmental delay<br>Growth delay<br>Increased serum lactate<br>Thick eyebrow	*FBXL4*	c.858+5G>C/<br>c.1510T>C	p=0.0032	Mitochondrial DNA depletion syndrome 13<br>#615471	No	Carriers/Affected brother<br>Autosomal recessive
44	5 y	M	Increased serum lactate<br>Infantile axial hypotonia<br>Ataxia<br>Severe expressive language delay<br>Short corpus callosum<br>Hypotonia<br>Seizures<br>Lactic acidosis	*FARS2*	c.1256G>A/<br><br>g.(?_5404749)_(5404959_?)del	p=0.0012	Combined oxidative phosphorylation deficiency 14<br>#614946	No	Carriers<br>Autosomal recessive
45	4 m	M	Increased serum lactate<br>Sudden cardiac death,<br>Encephalopathy	*ACAD9*	c.358delT/<br>c.809-2A>G	p=0.0018	Mitochondrial complex I deficiency, nuclear type 20	No	Carriers<br>Autosomal recessive

			Hypoglycemia<br>Acute liver failure				#611126		
46	12 y	M	Delayed psychomotor development<br>Metabolic acidosis	*MTO1*	c.170G>T/	p=0.4211	Combined oxidative phosphorylation deficiency 10<br>#614702	No	Carriers<br>Autosomal recessive
47	2 m	M	Lactic acidosis	*GFM1*	c.640A>T/<br>c.1199G>C	p=0.4194	Combined oxidative phosphorylation deficiency 1<br>#609060	No	Carriers<br>Autosomal recessive
48	1 y	M	Lactic acidosis<br>Nystagmus<br>Ragged-red muscle fibers<br>Global developmental delay<br>Failure to thrive in infancy	*COX15*	c.649C>T/<br>c.649C>T	p=0.0548	Mitochondrial complex IV deficiency, nuclear type 6<br>#615119	No	Carriers<br>Autosomal recessive
49	17 y	F	Leukodystrophy<br>Agenesis of corpus callosum<br>Decreased activity of mitochondrial complex I and III<br>Lactic acidosis<br>Increased CSF lactate	*EARS2*	c.670G>A/<br>c.376C>T	p=0.0013	Combined oxidative phosphorylation deficiency 12<br>#614924	No	Carriers<br>Autosomal recessive
50	12 y	M	Muscle weakness<br>Encephalopathy<br>Lactic acidosis	*ACAD9*	c.737T>C/<br>c.860G>A	p=0.0005	Mitochondrial complex I deficiency, nuclear type 20<br>#611126	No	Carriers/Affected brother<br>Autosomal recessive
51	-	M	Encephalopathy<br>Generalized hypotonia<br>Lactic academia<br>Ptosis<br>Generalized hypotonia<br>Failure to thrive<br>Global developmental delay	*C12orf65*	c.207_220del/<br>c.207_220del	p=0.0599	Combined oxidative phosphorylation deficiency 7<br>#613559	No	Carriers<br>Autosomal recessive
52	17 y	F	Hypotonia<br>Increased serum and CSF lactate	*PNPT1*	c.1177-2A>C/<br>c.1519G>T	p=0.0599	Combined oxidative phosphorylation deficiency 13<br>#614932	No	Carriers<br>Autosomal recessive
53	1 y	F	Nystagmus<br>Pigmentary retinopathy<br>Ptosis<br>Dystonia	*PET100*	c.1A>G	p=0.0055	Mitochondrial complex IV deficiency, nuclear type 12<br>#619055	No	Carriers<br>Autosomal recessive

			Global developmental delay<br>Respiratory insufficiency						
54	-	F	Hyperlacticaemia<br>Metabolic acidosis<br>Encephalopathy	*EARS2*	c.184A>T/<br>c.920T>C	p=0.0368	Combined oxidative phosphorylation deficiency 12<br>#614924	No	Carriers<br>Autosomal recessive
55	1 m	M	Neonatal hypotonia<br>Lethargy<br>Focal seizures<br>Diabetes insipidus<br>Cerebellar and cerebral cortical atrophy	*COQ4*	c.202G>C/<br>c.718C>T	p=0.2884	Coenzyme Q10 deficiency, primary, 7<br>#616276	Yes	Carriers<br>Autosomal recessive
56	1 m	F	Premature birth<br>Hyperlacticaemia<br>Intrauterine growth retardation<br>Oligohydramnios<br>Muscle weakness	*TMEM70*	c.317-2A>G/<br>c.317-2A>G	p=0.0417	Mitochondrial complex V (ATP synthase) deficiency, nuclear type 2<br>#614052	No	Carriers<br>Autosomal recessive
57	12 y	F	Epileptic encephalopathy<br>Status epilepticus<br>Clonus<br>Generalized hypotonia<br>Progressive spasticity	*ATPAF2*	c.133+1G>T/<br>c.133+1G>T	p=0.053	Mitochondrial complex V (ATP synthase) deficiency, nuclear type 1<br>#604273	No	Carriers<br>Autosomal recessive
58	2 m	M	Hyperlacticaemia<br>Methylmalonic aciduria<br>Mitochondrial DNA depletion<br>Encephalopathy	*SUCLA2*	c.850C>T	p=0.0375	Mitochondrial DNA depletion syndrome 5 (encephalomyopathic form with or without methylmalonic aciduria)<br>#612073	No	Carriers<br>Autosomal recessive
59	8 y	M	Spastic tetraparesis<br>Progressive encephalopathy<br>Abnormality of the gastrointestinal tract	*MCCC1*	c.1441C>A/<br>c.1423G>A	p=0.3542	3-Methylcrotonyl-CoA carboxylase 1 deficiency<br>#210200	Yes	Carriers<br>Autosomal recessive
60	1 m	M	Metabolic acidosis<br>Myocardial fibrosis<br>Reduced consciousness/confusion<br>Respiratory difficulties	*TK2*	c.230C>T/<br>c.31+470G>A	p=0.2884	Mitochondrial DNA depletion syndrome, myopathic form<br>#609560	No	Carriers<br>Autosomal recessive

** Changes in medication and/or diet after genetic diagnosis ;d:days; F: female; m: month; M: male; y:years*

Supplementary Table S4 - Clinical data and genetic findings for each patient for which a positive diagnosis was established using the COMP MOL panel.

**Cases**	**Age**	**Sex**	**HPO**	**Gene**	**Variants**	**Phenomizer**	**Disorder MIM**	**Changes in Medication or Diet***	**Parents/Inheritance**
61	-	F	Thrombocytopenia<br>Glaucoma<br>Nystagmus<br>Increased CSF interferon alpha	*TREX1*	c.218C>T/<br>c.404A>C	p=0.0525	Aicardi-Goutieres syndrome 1<br>#225750	No	Carriers, affected sister<br>Autosomal recessive
62	10 y	M	Specific learning disability<br>Ataxia<br>Cerebellar atrophy<br>EMG: axonal abnormality<br>Attention deficit/hyperactivity<br>Generalized myoclonic seizures<br>Global developmental delay	*TPP1*	c.622C>T/<br>c.887-10T>C	p=0.0187	Ceroid lipofuscinosis, neuronal, 2<br>#204500	Yes	Carriers<br>Autosomal recessive
63	5 y	F	Developmental regression<br>Epileptic encephalopathy<br>Apathy<br>Generalized hypotonia	*HEXA*	c.533G>A/<br>c.380T>G	p=0.0628	Tay-Sachs disease<br>#272800	No	Carriers<br>Autosomal recessive
64	15 y	M	Bilateral ptosis<br>Dysmetria<br>Dysarthria<br>Horizontal nystagmus<br>Progressive cerebellar ataxia<br>Learning disability	*POLR3B*	c.1244T>C/<br>c.2740G>A	p=0.0008	Leukodystrophy, hypomyelinating, 8, with or without oligodontia and/or hypogonadotropic hypogonadism<br>#614381	No	Carriers/Affected brother<br>Autosomal recessive
65	-	F	Increased neuronal autofluorescent lipopigment<br>Ataxia<br>Seizures	*PPT1*	c.541G>T/<br>c.541G>T	p=0.0411	Ceroid lipofuscinosis, neuronal, 1<br>#256730	No	Carriers<br>Autosomal recessive
66	5 y	M	Arthralgia<br>Joint stiffness	*GNPTAB*	c.1325G>A/<br>c.1325G>A	p=1.000	Mucolipidosis II alpha/beta<br>#252500	No	Carriers<br>Autosomal recessive

67	5 y	F	Progressive spasticity<br>Abnormality of peripheral nerve conduction<br>Developmental regression	*HSD17B4*	c.1273T>C/<br>c.1528G>A	p=0.1340	D-bifunctional protein deficiency<br>#261515	No	Carriers<br><br>Autosomal recessive
68	6 y	F	Encephalopathy<br>CNS demyelination<br>Generalized hypotonia<br>Delayed speech and language development<br>Seizures<br>Global developmental delay	*SLC17A5*	c.918T>G/<br>c.1239C>G	p=0.0339	Salla disease<br>#604369<br><br>Sialic acid storage disorder, infantile<br>#269920	No	Carriers<br><br>Autosomal recessive
69	8 m	M	Cerebral palsy<br>Bilateral sensorineural hearing impairment<br>Optic atrophy<br>Abnormal basal ganglia MRI signal intensity<br>Cerebellar atrophy<br>Peripheral axonal neuropathy<br>Seizures	*PLA2G6*	c.1460_1462del/<br>c.1460_1462del	p=0.0205	Neurodegeneration with brain iron accumulation 2B<br>#610217	No	Carriers<br><br>Autosomal recessive
70	3 y	M	Coarse facial features<br>Global developmental delay<br>Hyperactivity<br>Sensorial ataxia	*NAGLU*	c.531+5G>A/<br>c.1045_1047del	p=0.3261	Mucopolysaccharidosis type IIIB (Sanfilippo B)<br>#252920	No	Carriers<br><br>Autosomal recessive
71	7 y	M	Abnormality of cerebral white matter<br>Impaired smooth pursuit<br>Abnormal emotion/affect behavior<br>Vacuolated lymphocytes	*POLR3A*	c.2376_2377delAT/<br>c.2081G>A	p=0.489	Leukodystrophy, hypomyelinating, 7, with or without oligodontia and/or hypogonadotropic hypogonadism<br>#607694	No	Carriers<br><br>Autosomal recessive
72	4 y	F	Hepatosplenomegaly<br>Abnormality of blood and blood-forming tissues<br>Hyposmia<br>Pain<br>Hypersplenism	*GBA*	c.1226A>G/<br>c.930T>A	p=0.0804	Gaucher disease, type I<br>#230800	Yes	Carriers<br><br>Autosomal recessive
73	5 m	F	Seizures<br>Hypotonia	*PEX12*	c.888_889del/<br>c.888_889del	p=0.0055	Peroxisome biogenesis disorder 3	No	Carriers

			Increased very long chain fatty acids				#614859		Autosomal recessive
74	9 y	F	Motor deterioration<br>Fingerprint intracellular accumulation of autofluorescent lipopigment storage material	*CLN6*	c.829_836delinsCCT	p=0.0028	Ceroid lipofuscinosis, neuronal, 6<br>#601780	No	Carriers<br>Autosomal recessive
75	4 m	M	Hepatosplenomegaly<br>Muscular dystrophy<br>Generalized hypotonia	*CTSA*	c.1252G>T/<br>c.1252G>T	p=0.7652	Galactosialidosis<br>#256540	No	Carriers<br>Autosomal recessive
76	4 y	F	Macroglossia<br>Relative macrocephaly<br>Abnormality of mucopolysaccharide metabolism<br>Hepatosplenomegaly<br>Hearing impairment<br>Corpus callosum atrophy	*SGSH*	c.355+1G>A/<br>c.1080delC	p=0.4196	Mucopolysaccharidosis type IIIA<br>(Sanfilippo A)<br>#252900	No	Carriers<br>Autosomal recessive
77	7 m	F	Hepatomegaly<br>Elevated hepatic transaminases<br>Epicanthus<br>Broad forehead<br>Increased urinary O-linked Sialopeptides	*GNE*	c.797G>A	p=0.529	Sialuria<br>#269921	No	Affected sisters<br>Autosomal dominant
78	-	M	Delayed speech and language development<br>Hepatomegaly<br>Microcephaly<br>Umbilical hernia<br>Abnormality of joint mobility<br>Broad eyebrow	*SGSH*	c.1127T>C/<br>c.197C>G	p=0.5943	Mucopolysaccharidosis type IIIA<br>(Sanfilippo A)<br>#252900	No	Carriers<br>Autosomal recessive
79	1 y	M	Profound global developmental delay<br>Strabismus<br>Increased serum lactate<br>Abnormal CNS myelination<br>Increased CSF lactate	*SLC16A2*	c.46delC	p=0.4992	Allan-Herndon-Dudley syndrome<br>#300523	No	Mother<br>X-linked
80	5 y	M	Hepatosplenomegaly<br>Thrombocytopenia	*GBA*	c.1193G>A/<br>c.155C>T	p=0.2962	Gaucher disease, type I<br>#230800	Yes	Carriers<br>Autosomal

									recessive
81	10 y	F	Coarse facial features<br>Global developmental delay<br>Urinary glycosaminoglycan excretion<br>Hepatomegaly<br>Splenomegaly<br>Growth abnormality	*NAGLU*	c.1562C>T/<br>c.1693C>T	p=0.0425	Mucopolysaccharidosis type IIIB (Sanfilippo B)<br>#252920	No	Carriers<br>Autosomal recessive
82	6 y	M	Seizures<br>Developmental regression<br>Generalized hypotonia	*HEXA*	c.533G>A	p=0.3875	GM2-gangliosidosis<br>#272800	No	Carriers<br>Autosomal recessive
83	5 y	M	Muscular hypotonia<br>Cardiomyopathy<br>Elevated serum creatine phosphokinase	*GAA*	c.343C>T/<br>c.-32-13T>G	p=0.3778	Glycogen storage disease II<br>#232300	Yes	Carriers<br>Autosomal recessive

** Changes in medication and/or diet after genetic diagnosis ;d:days; F: female; m: month; M: male; y:years*

Supplementary Table S5 - Clinical data and genetic findings for each patient with a negative result on the MITO panel and subsequent positive diagnosis with the NeuroSeq panel. Highlighting indicates genes associated with metabolic disorders (orange) and neurological disorders (blue).

Cases	Age	Sex	HPO	Gene	Variants	Phenomizer	Disorder	MIM	Parents/Inheritance	Allocated Panel
84	2 y	M	Dilated cardiomyopathy<br>Blindness<br>Nystagmus	*ALMS1*	c.7586C>G/<br>c.11017C>T	p=0.2338	Alstrom syndrome	#203800	Carriers<br>Autosomal recessive	**HYPO/HYPER**
85	10 y	M	Decreased activity of mitochondrial complex I	*SLC16A2*	c.99dupT	p=1.0000	Allan-Herndon-Dudley syndrome	#300523	De novo<br>X-linked dominant	**COMP MOL**
86	5 m	M	Spastic tetraparesis<br>Polyneuropathy<br>Developmental regression<br>Generalized hypotonia<br>Global developmental delay	*POLR3A*	c.2248-1G>C/<br>c.1771-7C>G	p=0.0266	Wiedemann-Rautenstrauch syndrome	#264090	Carriers<br>Autosomal recessive	**COM MOL**
87	2 m	M	Increased serum lactate<br>Encephalopathy<br>Seizures	*LIPT1*	c.212C>T/<br>c.292C>T	p=0.2631	Lipoyltransferase 1 deficiency	#616299	Carriers<br>Autosomal recessive	**MITO updated**
88	2 y	F	Hypoglycemia<br>Lactic acidosis<br>Thin vermilion border<br>Abnormal facial shape<br>Widely spaced teeth	*LRPPRC*	c.1678A>T/<br>c.1736-7T>G	p=0.4006	Mitochondrial complex IV deficiency, nuclear type 5	#220111	Carriers<br>Autosomal recessive	**MITO updated**
89	7 y	M	Epilepsia partialis continua<br>Epileptic encephalopathy<br>Mitochondrial depletion	*KCNT1*	c.785G>A/<br>c.3503-1G>A	p=0.0195	Epilepsy, nocturnal frontal lobe, 1	#615005	Father/*De novo*<br>Autosomal dominant	**EPILEPSY**
90	3 y	F	Encephalopathy<br>Dystonia<br>Decreased activity of mitochondrial complex I<br>Ragged-red muscle fibers	*GNAO1*	c.607G>A	p=0.064	Developmental and epileptic encephalopathy 17	#615473	*De novo*<br>Autosomal dominant	**EPILEPSY**
91	4 y	M	Generalized hypotonia<br>Muscular hypotonia<br>Epileptic encephalopathy	*KCNQ2*	c.629G>A	p=0.0027	Developmental and epileptic encephalopathy 7	#613720	*De novo*<br>Autosomal dominant	**EPILEPSY**

			Seizures<br>Global developmental delay							
92	3 y	F	Encephalopathy<br>Lactic acidosis<br>Developmental regression<br>Dystonia<br>Psychomotor deterioration<br>Abnormality of body weight	*GNAO1*	c.709G>A	p=0.0383	Developmental and epileptic encephalopathy 17	#615473	*De novo*<br><br>Autosomal dominant	**EPILEPSY**
93	2 y	F	Epileptic encephalopathy<br>Cortical atrophy<br>Progressive leukoencephalopathy	*KCNQ2*	c.833T>C	p=0.0383	Developmental and epileptic encephalopathy 7	#613720	*De novo*<br><br>Autosomal dominant	**EPILEPSY**
94	4 m	M	Hypotonia<br>Diffuse hypomyelination	*SPTAN1*	c.6910_6918dup CAGCTGGGC	-	Developmental and epileptic encephalopathy 5	#613477	*De novo*<br><br>Autosomal dominant	**EPILEPSY**
95	7 y	M	Ophthalmoplegia	SBF2	c.2380G>T/<br>c.2380G>T	p=0.3379	Charcot-Marie-Tooth disease, Type 4b2	#604563	Carriers<br><br>Autosomal recessive	**NEUROMUSCULAR DISORDER**
96	18 y	M	Peripheral neuropathy<br>Ataxia<br>Pigmentary retinopathy	*FLVCR1*	c.721G>A/<br>c.721G>A	p=0.2157	Ataxia, posterior column, with retinitis pigmentosa	#609033	Carriers<br><br>Autosomal recessive	***MOVEMENT DISORDERS***
97	5 y	F	Abnormality of coordination<br>Delayed speech<br>Language development	*STAG1*	c.2285T>A	-	Mental retardation, autosomal dominant 47	#617635	*De novo*<br><br>Autosomal dominant	***INTELLECTUAL DISABILITY***

d:days; F: female; m: month; M: male; NA: not available; y:years

Supplementary Table S6 - Clinical data and genetic findings for each patient for which an inconclusive diagnosis was obtained. Highlighting indicates clinical cases in which pathogenic variants (dark grey) and variants of uncertain significance (light grey) were detected.

Cases	Age	Sex	HPO	Panel	Gene	Variants	Disorder	Inheritance	Family Study
					**DOMINANT GENES**				
98	9 y	F	Hypoglycemia<br>Focal seizures<br>Hyperinsulinism	HYPO/HYPER	*GLUD1*	c.272C>T	Hyperinsulinism-hyperammonemia syndrome	AD	MOTHER<br>(maternal family history)
99	5 y	M	Decreased activity of mitochondrial complex IV<br>Seizures	MITO	*LETM1*	c.709C>T	Wolf-Hirschhorn Syndrome	AD	MOTHER<br>(maternal family history)
100	3 y	M	Hyperinsulinemia<br>Hypoglycemia	COM MOL	*KCNJ11*	c.1105C>A	Hyperinsulinemia Hypoglycemia, familial	AD	NA
101	18 y	F	Hyperglycemia<br>Maturity-onset diabetes of the young	HYPO/HYPER	*HNF1A*	c.1295 C>T	MODY type II	AD	NA
102	4 y	M	Hyperalaninemia<br>Hyperglutaminemia<br>Increased serum lactate<br>Asymmetric septal hypertrophy<br>Hypertrophic cardiomyopathy<br>Strabismus<br>Encephalopathy<br>Global developmental delay<br>Increased CSF lactate<br>Seizures	NeuroSeq	*WAC*	c.1583C>T	Desanto-Shinawi syndrome	AD	NA
103	-	F	Macrocephaly<br>Hypoglycemia<br>Metabolic acidosis<br>Global developmental delay	INT MET	*IDH2*	c.419G>A	D-2-hydroxyglutaric aciduria 2	AD	NA

104	5 y	M	Mitochondrial depletion<br>Global developmental delay<br>Epilepsia partialis continua<br>Generalized hypotonia	NeuroSeq	*ZEB2*	c.3202_3207delGG CTCG	Mowat-Wilson syndrome due to monosomy 2q22	AD	NA
105	2 y	M	Decreased mitochondrial complex I and II activity	NeuroSeq	*ARID1B*	c.2281+1G>C	Coffin-Siris syndrome	AD	NA
106	-	F	Dysmyelinating leukodystrophy<br>Global developmental delay	COM MOL	*SOX10*	c.239T>C	Waardenburg syndrome type 2	AD	NA
107	10 y	M	Profound global developmental delay<br>Gastroesophageal reflux<br>Ventriculomegaly<br>Agenesis of corpus callosum<br>Microcephaly<br>Upper airway obstruction<br>Dysphagia<br>Pontocerebellar atrophy	COM MOL	*LMNB1*	c.1025_1026delAA	Leukodystrophy, adult-onset, autosomal dominant	AD	NA
108	2 y	M	Recurrent bacterial infections<br>Generalized hypotonia<br>Mild receptive language delay	NeuroSeq	*ZNF41*	c.2057T>C	-	XL	NA
109	5 y	F	Ketotic hypoglycemia<br>Episodic ketoacidosis	HYPO/HYPER	*PHKA2*	c.2911C>T	Glycogen storage disease, type IX	XLD	NA
110	6 y	F	Seizures<br>Global developmental delay	NeuroSeq	*PDHA1*	c.577_578delTT	Pyruvate dehydrogenase E1-alpha deficiency	XL	NA
111	5 y	M	Decreased activity of mitochondrial complex IV<br>Seizures	MITO	*NDUFB11*	c.152C>A	?Mitochondrial complex I deficiency, nuclear type 30	XL	MOTHER
				**RECESSIVE GENES**					
112	3 y	M	Epileptic encephalopathy<br>Abnormal CSF lactate level<br>Cerebral cortical atrophy<br>Leukoencephalopathy	MITO	*POLRMT*	c.3596T>C	? Autosomal dominant progressive external ophthalmoplegia and mitochondrial DNA depletion syndrome	-	TRANS

							4A		
113	6 y	M	NA	HYPO/HYPER	*ETFDH*	c.1274C>G	Glutaric acidemia IIC	AR	NA
114	2 y	F	Celiac disease<br>Spastic diplegia<br>Spastic paraparesis<br>Leukoencephalopathy	COM MOL	*RNASEH2B*	c.655T>C	Aicardi-Goutieres Syndrome 2	AR	NA
115	9 y	M	Hyperornithinemia<br>Hydroxyprolinemia<br>Hydroxyprolinuria	INT MET	*PRODH*	c.1397C>T	Hyperprolinemia, type I	AR	MOTHER
116	9 y	M	Fatigue<br>Recurrent infections<br>Spinal rigidity	MITO	*DARS2*	c.396+2T>G	Leukoencephalopathy with brain stem and spinal cord involvement and lactate elevation	AR	NA
117	-	F	Global developmental delay<br>Epileptic encephalopathy<br>Postnatal microcephaly	COM MOL	*PPT1*	c.2T>C	Ceroid lipofuscinosis, neuronal, 2	AR	MOTHER
118	9 y	M	Dysphagia<br>Optic neuritis<br>Ptosis<br>Strabismus<br>Fatigable weakness<br>Hypertonia<br>Mitochondrial encephalopathy<br>Increased CSF lactate	MITO	*BCS1L*	c.166C>T	Mitochondrial complex III deficiency, nuclear type 1	AR	FATHER
119	3 m	M	Orotic aciduria	INT MET	*UMPS*	c.1213C>T	Orotic aciduria	AR	NA
120	11 y	M	Lactic acidosis<br>Decreased activity of cytochrome C oxidase in muscle tissue<br>Cerebral palsy	MITO	*PNPT1*	c.493C>T	Combined oxidative phosphorylation deficiency 13	AR	MOTHER
121	12 y	M	Decreased activity of mitochondrial complex I	MITO	*ADCK4*	c.103G>A	Nephrotic syndrome, type 9 (coenzyme Q10 deficiency)	AR	MOTHER
122	8 y	F	Decreased activity of mitochondrial complex II<br>Decreased activity of mitochondrial	MITO	*NDUFAF2*	c.490delG	Mitochondrial complex I deficiency, nuclear type 10	AR	MOTHER

			complex IV<br>Cognitive impairment						
123	-	F	Seizures<br>Global developmental delay	COM MOL	*CLN6*	c.314C>T	Ceroid lipofuscinosis, neuronal, 6	AR	NA
124	5 y	M	Focal autonomic seizures with altered responsiveness<br>Mild microcephaly<br>Central apnea<br>Moderate postnatal growth retardation<br>Generalized hypotonia<br>Hypertelorism<br>Wide nose<br>Anemia<br>Global developmental delay	NeuroSeq	*MTO1*	c.1390C>T	Combined oxidative phosphorylation deficiency 12	AR	NA
125	17 y	F	Ketotic hypoglycemia<br>Episodic ketoacidosis<br>Febrile seizures<br>Global developmental delay	HYPO/HYPER	*PHKA2*	c.2870A>G	Glycogen storage disease, type IX	XLR	MOTHER
126	-	M	Decreased activity of mitochondrial complex I<br>Decreased activity of mitochondrial complex II and III	MITO	*SLC6A8*	c.928G>A	Cerebral creatine deficiency syndrome 1	XLR	NA

DOMINANT AND RECESSIVE INHERITANCE

127	8 y	F	Decreased activity of mitochondrial complex II<br>Decreased activity of mitochondrial complex IV<br>Pyramidal syndrome	MITO	*DNM1L*	c.1921G>A	Encephalopathy, lethal, due to defective mitochondrial peroxisomal fission 1	AD, AR	NA
128	2 y	M	Noninsulin-dependent diabetes mellitus	HYPO/HYPER	*PDX1*	c.779G>A	MODY, type IV	AD, AR	FATHER<br>(paternal family history)
129	-	M	Hyperinsulinemia<br>Hypoglucemia+<br>macrosomia	HYPO/HYPER	*ABCC8*	g.(?_17464699)_(17464884_?)del<br>g.(?_17464241)_(17464454_?)del	Familial hyperinsulinemic hypoglycemia	AD, AR	NA

130	1 y	M	Abnormality of coordination<br>Gait disturbance	MITO	*SPG7*	c.1529C>T	Spastic Paraplegia Type 7	AD, AR	NA
**POSSIBLE CASE OF DIGENIC INHERITANCE**									
131	1 y	F	Ketotic hypoglycemia<br>Compensated hypothyroidism	HYPO/HYPER	*ABCC8*<br>*SLC37A4*	c.3004G>A<br>c.1225A>G/ c.1022C>T	Hypoglycemia of infancy, leucine-sensitive<br>Glycogen storage disease	AD, AR<br>AR	*DE NOVO*<br>TRANS

d:days; F: female; m: month; M: male; NA: not available; y:years

Supplementary Table S7 – Design of five different multi-gene panels consisting of a group of genes previously associated with: inborn errors of intermediary metabolism (INT MET), hypoglycemic/hyperglycemic events (HYPO/HYPER) associated with metabolic disorders or other processes, mitochondrial diseases (MITO), complex molecular defects including leukodystrophies (COMP MOL), and NeuroSeq panel which includes all the genes from the individual panels, along with genes associated with neurological disorders.

INT MET panel

ABCD4	ACADM	ACADS	ACADSB	ACADVL	ACAT1	ACAT2	ACSF3	ADA	ADK	ADSL	AGL	AGXT	AGXT2	AHCY	ALDH4A1		ALDH5A1
ALDH6A1		ALDOB	AMT	APRT	ARG1	ASL	ASS1	AUH	BCKDHA		BCKDHB	BTD	C2ORF25	C7ORF10	CBS	CD320	CLPB
CLPX	CPS1	CPT1A	CPT2	CTH	D2HGDH		DBT	DHTKD1	DLD	DNAJC12	DNAJC19	DPYD	DPYS	ETFA	ETFB	ETFDH	ETHE1
FAH	FBP1	FH	FMO3	FTCD	G6PC	GALE	GALK1	GALT	GBE1	GCDH	GCH1	GCSH	GGT1	GLDC	GLUL	GLYCTK	GNMT
GPHN	GRHPR	GSTZ1	GYS2	HADHA	HADHB	HAL	HCFC1	HGD	HLCS	HMGCL	HMGCS2	HOGA1	HPD	HPRT1	HSD17B10		HTRA2
IDH2	IVD	L2HGDH	LMBRD1	MAT1A	MCCC1	MCCC2	MCEE	MDH1	MLYCD	MMAA	MMAB	MMACHC		MMADHC		MOCOS	MOCS1
MOCS2	MTHFR	MTR	MTRR	MUT	NAGS	NT5C3A	OAT	OGDH	OPA3	OTC	OXCT1	PAH	PC	PCBD1	PCCA	PCCB	PCK1
PCK2	PEPD	PGM1	PHKA2	PHKB	PHKG2	PKLR	PMM2	PNP	PRODH	PRPS1	PTS	PYGL	QDPR	SERAC1	SLC1A1	SLC22A5	SLC25A1
SLC25A13		SLC25A15		SLC25A20		SLC2A2	SLC37A4	SLC46A1	SLC52A1	SLC6A19	SLC6A9	SUCLA2	SUCLG1	SUOX	TAT	TAZ	TCN2
TIMM50	UMPS	UPB1	XDH														

HYPO/HYPER panel

ABCC8	AKT2	ALMS1	APPL1	ASXL2	BLK	BSCL2	CAPN10	CDKAL1	CEL	DIS3L2	ENPP1	GCGR	GCK	GLIS3	GLUD1	GPD2	HADH
HMGA1	HNF1A	HNF1B	HNF4A	IGF2BP2	IL6	INS	INSR	IRS1	IRS2	KCNJ11	KLF11	LEP	LEPR	LIPC	LMNA	MAFA	MAPK8IP1
MC4R	MEN1	MPI	MTNR1B	NEUROD1		NSD1	PAX4	PCSK1	PDX1	PGM1	PLAGL1	PLIN1	PLUT	POMC	PPARA	PPARG	PPP1R3A
PTPN1	PYGL	RETN	SH2B1	SIM1	SLC16A1	SLC2A1	SLC2A2	SLC30A8	SLC5A1	TCF2	TCF7L2	UBE3B	UCP2	UMOD	WFS1	ZFP57	

MITO panel

AARS2	ABCB11	ACAD8	ACAD9	ACO2	ADCK1	ADCK2	ADCK3	ADCK4	ADCK5	ADSL	AFG3L2	AGK	AIFM1	AKR1B15	APOPT1	APTX	ATAD1
ATAD3A	ATP5A1	ATP5B	ATP5C1	ATP5D	ATP5E	ATP5F1	ATP5F1D	ATP5G1	ATP5G2	ATP5G3	ATP5O	ATPAF1	ATPAF2	BCS1L	BOLA3	C10ORF2	C12ORF65
C19ORF12		C1QBP	CA5A	CARS2	CCDC88A		CEP89	CHCHD10		CLPB	CLPP	COA5	COA6	COA7	COQ10A	COQ10B	COQ2
COQ3	COQ4	COQ5	COQ6	COQ7	COQ9	COX10	COX11	COX14	COX15	COX16	COX17	COX18	COX19	COX4I1	COX4I2	COX6A1	COX6A2
COX6B1	COX6B2	COX7B	COX8A	CPS1	CYC1	CYCS	DARS2	DEAF1	DGUOK	DLAT	DLD	DNA2	DNAJC19	DNM1L	E4F1	EARS2	ECHS1
ELAC2	ETHE1	FAM36A	FARS2	FARSB	FASTKD2		FBXL4	FDX1L	FH	FLAD1	FOXRED1		GARS	GFER	GFM1	GFM2	GLRX5
GOT2	GPT2	GTPBP3	GYG2	HARS2	HCCS	HIBCH	HSD17B10		HSD17B4	HSPD1	HSPE1	HTRA2	IARS	IARS2	IBA57	ISCA1	ISCA2
ISCU	ITPA	KARS	LARS2	LETM1	LGI1	LIAS	LIPT1	LIPT2	LONP1	LRP4	LRPPRC	LYRM4	LYRM7	MARS2	MDH2	MECP2	MFF
MFN1	MFN2	MGME1	MICU1	MIPEP	MNF1	MPV17	MRPL12	MRPL3	MRPL44	MRPS16	MRPS22	MRPS23	MRPS34	MRPS7	MTCH1	MTERF1	MTFMT
MTHFD1L		MTO1	MTPAP	NADK2	NARS2	NAT8L	NAXE	NDUFA1	NDUFA10		NDUFA11		NDUFA12		NDUFA13		NDUFA2

NDUFA3 NDUFA4 NDUFA4L2 NDUFA5 NDUFA9 NDUFAF1 NDUFAF2 NDUFAF3 NDUFAF4 NDUFAF5 NDUFAF6
NDUFB1 NDUFB10 NDUFB11 NDUFB2 NDUFB3 NDUFB4 NDUFB5 NDUFB6 NDUFB7 NDUFB8 NDUFB9 NDUFS1 NDUFS2 NDUFS3 NDUFS4 NDUFS5
NDUFS6 NDUFS7 NDUFS8 NDUFV1 NDUFV2 NDUFV3 NFS1 NFU1 NUBPL OPA1 OPA3 PARS2 PC PCK2 PDHA1 PDHA2 PDHB PDHX
PDK3 PDP1 PDP2 PDSS1 PDSS2 PET100 PET117 PGAP2 PIGN PMPCB PNPLA4 PNPLA8 PNPT1 POLG POLG2 POLRMT PTCD1 PUS1
QRSL1 RANBP2 RARS2 RERE RMND1 RNASEH1 RPIA RRM2B RTN4IP1 SARS SARS2 SCO1 SCO2 SDHA SDHAF1 SDHAF2 SDHB
SDHC SDHD SERAC1 SFXN4 SLC19A3 SLC25A12 SLC25A19 SLC25A26 SLC25A3 SLC25A4 SLC25A42 SLC25A46 SLC52A2
SLC6A8 SLC6A9 SPG20 SPG7 SUCLA2 SUCLG1 SURF1 TACO1 TANGO2 TARS2 TAZ TAZ TBCD TBCE TFAM TIMM8A TK2 TKT
TMEM126A TMEM126B TMEM70 TOP3A TPK1 TRAK1 TRIT1 TRMT10C TRMT5 TRMU TRNT1 TSFM TTC19 TUBB TUBB6
TUFM TXN2 TYMP UPB1 UQCC3 UQCR10 UQCR11 UQCRB UQCRC1 UQCRC2 UQCRFS1 UQCRH UQCRHL UQCRQ VAC14 VARS2 VDAC1
VDAC2 WARS2 YARS2 YME1L1

COMP MOL panel

AARS AARS2 ABCD1 ABCD3 ABHD12 ABHD5 ACAA1 ACER3 ACOX1 ADAR AGA AGPS AIFM1 AIMP1 AIMP2 AKR1D1 ALDH3A2
AMACR APOPT1 ARSA ARSB ASAH1 ASPA ATP13A2 ATRN BCAP31 C11ORF73 C19ORF12 CLCN2 CLN3 CLN5 CLN6 CLN8
CNTNAP1 COL4A1 CP CRAT CSF1R CTBP1 CTC1 CTSA CTSD CTSF CYP27A1 CYP7B1 DARS DHCR24 DHCR7 DNAJC5 DYM
EBP EIF2B1 EIF2B2 EIF2B3 EIF2B4 EIF2B5 EPRS ERAL1 FA2H FAM126A FDFT1 FUCA1 GAA GALC GALNS GBA GBE=GBE1
GFAP GJC2 GLA GLB1 GM2A GM1 GNE GNPAT GNPTAB GNPTG GNS GPHN GRN GUSB HEPACAM HEXA HEXB
HGSNAT HSD17B4 HSD3B7 HSPD1 HTRA1 HYAL1 IBA57 IDS IDUA IFIH1 ITPA KCNT1 KCTD7 LAMP2 LIPA LMNB1 MAG MAN2B1
MANBA MCOLN1 MFSD8 MLC1 MSMO1 MVK NAGA NAGLU NALCN NANS NDUFA2 NEU1 NKX6-2 NOTCH3 NPC1 NPC2 NPL NSDHL
NUBPL PEX1 PEX10 PEX11B PEX12 PEX13 PEX14 PEX16 PEX19 PEX2 PEX26 PEX3 PEX5 PEX6 PEX7 PHYH PLEKHG2
PLP1 PMM2 POLR1A POLR1C POLR3A POLR3B PPT1 PSAP PYCR2 RAB33B RARS REPS1 RNASEH2A RNASEH2B RNASEH2C
RNASET2 SAMHD1 SC5D SCARB2 SCP2 SGSH SIGMAR1 SLC16A2 SLC17A5 SLC1A4 SLC25A12 SMPD1 SNORD118 SOX10
SPTAN1 SUMF1 TBCD TMEM106B TPP1 TREX1 TUBB4A UBTF UFM1 VPS11 VPS33A

NeuroSeq panel

AARS AARS2 AASS ABAT ABCB11 ABCB7 ABCC8 ABCD1 ABCD3 ABCD4 ABHD12 ABHD5 ACAA1 ACAD8 ACAD9 ACADM ACADS ACADSB
ACADVL ACAT1 ACAT2 ACBD5 ACER3 ACHE ACMSD ACO2 ACOX1 ACSF3 ACSL4 ACTA1 ACTB ACTG1 ADA ADAM22 ADAMTS2
ADAR ADAT3 ADCK1 ADCK2 ADCK3 ADCK4 ADCK5 ADCY5 ADCY6 ADD3 ADGRG1 ADGRG6 ADGRV1 ADK ADNP ADPRHL2 ADSL
ADSSL1 AEBP1 AFF2 AFG3L2 AGA AGK AGL AGMO AGO1 AGPS AGRN AGXT AGXT2 AHCY AHDC1 AHI1 AIFM1 AIMP1
AIMP2 AKR1B15 AKR1D1 AKT1 AKT2 AKT3 ALAD ALAS2 ALDH18A1 ALDH3A2 ALDH4A1 ALDH5A1 ALDH6A1
ALDH7A1 ALDOA ALDOB ALG1 ALG11 ALG12 ALG13 ALG14 ALG2 ALG3 ALG6 ALG8 ALG9 ALMS1 ALPK3 ALS2 ALX4
AMACR AMPD1 AMPD2 AMT ANK3 ANKLE2 ANKRD11 ANO10 ANO3 ANO5 AP1S2 AP3B2 AP4B1 AP4E1 AP4M1 AP4S1 AP5Z1
APBB1 APOA1BP APOL2 APOL4 APOPT1 APPL1 APRT APTX ARCN1 ARF1 ARFGEF2 ARG1 ARHGEF10 ARHGEF15 ARHGEF2
ARHGEF6 ARHGEF9 ARID1A ARID1B ARL13B ARL3 ARL6 ARL6IP1 ARMC4 ARMC9 ARSA ARSB ARSI ARV1 ARX ASAH1
ASCC1 ASH1L ASL ASNS ASPA ASPM ASS1 ATAD1 ATAD3A ATAD3B ATAD3C ATCAY ATG5 ATL1 ATL3 ATM ATP13A2 ATP1A2
ATP1A3 ATP1B1 ATP2A1 ATP2B3 ATP5A1 ATP5B ATP5C1 ATP5D ATP5E ATP5F1 ATP5F1D ATP5G1 ATP5G2 ATP5G3 ATP5O ATP6AP2 ATP6V0A2
ATP6V1A ATP7A ATP7B ATP8A2 ATPAF1 ATPAF2 ATR ATRN ATRX AUH AUTS2 B3GALNT2 B3GNT1 B4GALNT1 B4GALT1

B4GALT7 B9D1 B9D2 BAG3 BBIP1 BBS1 BBS10 BBS12 BBS2 BBS4 BBS5 BBS7 BBS9 BCAP31 BCKDHA BCKDHB BCKDK
BCL11A BCL11B BCS1L BEAN1 BICD2 BIN1 BLK BMP1 BOLA3 BPTF BRAF BRAT1 BRF1 BRPF1 BRWD3 BSCL2 BTD BVES
C6ORF66 C10ORF2 C11ORF73 C12ORF65 C19ORF12 C2ORF86 C5ORF42 C7ORF10 C8ORF37 CA5A CA8 CACNA1A
CACNA1B CACNA1C CACNA1D CACNA1G CACNA1H CACNA1S CACNA2D1 CACNA2D2 CACNB4 CACNG2
CAD CAMK2A CAMK2B CAMTA1 CAPN1 CAPN3 CARS2 CASC5 CASK CASQ1 CASR CAV3 CBL CBS CC2D1A
CC2D2A CCDC115 CCDC174 CCDC28B CCDC78 CCDC88A CCDC88C CCM1 CCND2 CCT5 CD320 CDH15 CDK13 CDK5 CDK5RAP2
CDK6 CDKL5 CDON CEL CENPE CENPF CENPJ CEP104 CEP120 CEP135 CEP152 CEP164 CEP290 CEP41 CEP63 CEP89 CERS1 CFL2
CHAMP1 CHAT CHCHD10 CHD1 CHD2 CHD7 CHD8 CHI3L1 CHKB CHMP1A CHMP1B CHRNA1 CHRNA2 CHRNA4 CHRNB1 CHRNB2 CHRND
CHRNE CHRNG CHST14 CIC CIT CIZ1 CLCN1 CLCN2 CLCN4 CLCN6 CLIC2 CLN3 CLN5 CLN6 CLN8 CLP1 CLPB
CLPP CLPX CLTC CNKSR2 CNNM2 CNOT3 CNPY3 CNTN1 CNTN2 CNTNAP1 CNTNAP2 COA5 COA6 COA7 COASY COG1
COG2 COG4 COG5 COG6 COG7 COG8 COL12A1 COL13A1 COL18A1 COL1A1 COL1A2 COL3A1 COL4A1 COL4A2 COL4A3BP COL5A1 COL5A2
COL5A3 COL6A1 COL6A2 COL6A3 COL6A6 COLEC11 COLQ COMT COQ10A COQ10B COQ2 COQ3 COQ4 COQ5 COQ6 COQ7 COQ9
COX10 COX11 COX14 COX15 COX16 COX17 COX18 COX19 COX4I1 COX4I2 COX6A1 COX6A2 COX6B1 COX6B2 COX7B COX8A CP CPA6
CPLX1 CPOX CPS1 CPT1A CPT1C CPT2 CRADD CRAT CRBN CRTAP CRYAB CSDE1 CSF1R CSNK1G1 CSNK2B CSPP1 CSTB
CTBP1 CTC1 CTCF CTH CTNNA2 CTNNB1 CTSA CTSD CTSF CTTNBP2 CTU2 CUL4B CUX2 CWF19L1 CXORF56 CYC1 CYCS
CYFIP2 CYP26B1 CYP27A1 CYP2U1 CYP7B1 D2HGDH DAB1 DAG1 DAO DAOA DARS DARS2 DBT DCAF17 DCAF8 DCC
DCHS1 DCX DDC DDHD1 DDHD2 DDOST DDX3X DEAF1 DENND5A DEPDC5 DES DGAT2 DGUOK DHCR24 DHCR7 DHDDS DHFR
DHTKD1 DHX30 DIAPH1 DISC1 DISP1 DLAT DLD DLG3 DLGAP4 DLL1 DMD DNA2 DNAJB2 DNAJB6 DNAJC12 DNAJC19 DNAJC3 DNAJC5
DNM1 DNM1L DNM2 DNMT1 DNMT3A DOCK3 DOCK7 DOCK8 DOK7 DOLK DPAGT1 DPM1 DPM2 DPM3 DPP6 DPYD DPYS
DRD3 DRD4 DRD5 DRP2 DSCAM DST DSTYK DYM DYNC1H1 DYRK1A DYSF E4F1 EARS2 EBF3 EBP ECEL1 ECHS1
EDC3 EEF1A2 EEF2 EFHC1 EFNB1 EFTUD2 EGR2 EIF2AK2 EIF2B1 EIF2B2 EIF2B3 EIF2B4 EIF2B5 EIF2S3 EIF4E ELAC2 ELOVL4 ELOVL5
ELP2 EMD EMILIN1 EML1 EMX2 ENTPD1 EOMES EPB41L1 EPM2A EPRS EPT1 ERAL1 ERBB3 ERBB4 ERF ERGIC1 ERLIN1 ERLIN2
ERMARD ETFA ETFB ETFDH ETHE1 EVL EXOSC3 EXOSC9 EXT2 EZH2 FA2H FAH FAM111B FAM126A FAM36A
FARS2 FARSB FAS FASTKD2 FAT2 FAT4 FBN1 FBN2 FBP1 FBXL4 FBXO11 FBXO31 FDFT1 FDX1L FECH FGD1 FGD4
FGF12 FGF14 FGF8 FGFR1 FGFR2 FGFR3 FH FHL1 FIG4 FKBP10 FKBP14 FKRP FKTN FLAD1 FLNA FLNC FLVCR1 FMN2
FMO3 FMR1 FOLR1 FOXA2 FOXG1 FOXH1 FOXP1 FOXP2 FOXRED1 FREM1 FRMD4A FRMPD4 FRRS1L FTCD FTL FTSJ1 FUCA1 FUS
FUT8 G6PC GAA GABBR2 GABRA1 GABRA5 GABRB1 GABRB2 GABRB3 GABRD GABRG2 GAD1 GALC GALE GALK1 GALNS GALT GAN
GARS GAS1 GATAD2B GBA GBA2 GBE1 GCDH GCH1 GCK GCSH GDAP1 GDI1 GEMIN4 GFAP GFER GFM1 GFM2
GFPT1 GGT1 GJB1 GJC2 GLA GLB1 GLDC GLDN GLE1 GLI2 GLI3 GLIS2 GLIS3 GLRA1 GLRB GLRX5 GLUD1 GLUL
GLYCTK GM2A GMPPA GMPPB GNAL GNAO1 GNAQ GNB1 GNB4 GNB5 GNE GNMT GNPAT GNPTAB GNPTG GNS GOLGA2 GOSR2
GOT2 GPAA1 GPC3 GPHN GPR88 GPRASP1 GPSM2 GPT2 GRB14 GRHPR GRIA3 GRIA4 GRID2 GRIK2 GRIN1 GRIN2A GRIN2B
GRIN2D GRM1 GRM5 GRN GSTZ1 GTPBP3 GUF1 GUSB GYG1 GYG2 GYS1 GYS2 GZF1 HACD1 HACE1 HADH HADHA HADHB
HAL HARS HARS2 HBA1 HCCS HCFC1 HCN1 HCN2 HCN4 HDAC8 HECW2 HEPACAM HERC2 HESX1 HEXA HEXB HFE
HGD HGSNAT HIBCH HINT1 HINT3 HIVEP2 HK1 HLCS HMBS HMGCL HMGCS2 HNF1A HNF4A HNF6 HNMT HNRNPA1 HNRNPA2B1
HNRNPDL HNRNPH1 HNRNPH2 HNRNPU HOGA1 HOXA1 HPCA HPD HPRT1 HRAS HSD17B10 HSD17B4 HSD3B7
HSPB1 HSPB3 HSPB8 HSPD1 HSPE1 HSPG2 HTR2A HTRA1 HTRA2 HUWE1 HYAL1 HYOU1 IARS IARS2 IBA57 ICK IDH2 IDS
IDUA IER3IP1 IFIH1 IFITM5 IFT122 IFT27 IFT64 IGBP1 IGHMBP2 IKBKAP IL11RA IL1RAPL1 IMPA1 INAVA INF2 INPP5E
INPP5K INS INSR INVS IQCB1 IQSEC1 IQSEC2 IRF2BPL ISCA1 ISCA2 ISCU ISPD ITGA5 ITGA7 ITPA ITPR1 IVD JPH1
KANK1 KARS KAT6A KATNB1 KBTBD13 KCNA1 KCNA2 KCNB1 KCNC1 KCNC3 KCND3 KCNH1 KCNH5 KCNJ10 KCNJ11 KCNMA1 KCNQ2
KCNQ3 KCNQ5 KCNT1 KCNT2 KCTD17 KCTD7 KDM5B KDM5C KDM6A KIAA0196 KIAA0226 KIAA0556 KIAA0586 KIAA1109

KIAA1279		KIAA2022		KIDINS220		KIF11	KIF14	KIF1A	KIF1B	KIF1C	KIF22	KIF2A	KIF4A	KIF5A	KIF5C	KIF7	KIRREL3
KLC2	KLF11	KLHL15	KLHL40	KLHL41	KMT2B	KMT5B	KPTN	KRAS	KY	L1CAM	L2HGDH	LAMA2	LAMB1	LAMB2	LAMC3	LAMP2	LARGE
LARS2	LAS1L	LDB3	LDHA	LEPRE1	LETM1	LGI1	LGI4	LIAS	LIMS2	LINGO1	LINS	LIPA	LIPT1	LIPT2	LITAF	LMAN2L	LMBRD1
LMNA	LMNB1	LMNB2	LMOD3	LNPK	LONP1	LRP2	LRP4	LRPPRC	LRRC56	LRSAM1	LTC4S	LYRM4	LYRM7	LZTFL1	LZTR1	MAFA	MAG
MAGEL2	MAGI2	MAN1B1	MAN2B1	MANBA	MAOA		MAP2	MAPK10	MAPT	MARS	MARS2	MASP1	MAT1A	MAT2A	MATR3	MBD5	MBOAT7
MCCC1	MCCC2	MCEE	MCM3AP		MCOLN1	MCPH1	MDH1	MDH2	MECP2	MECR	MED12	MED13L	MED17	MED20	MED23	MED25	MEF2C
MEGF10	MEIS2	METTL23	MFF	MFN1	MFN2	MFSD2A	MFSD8	MGAT2	MGME1	MICU1	MID2	MIPEP	MIR17HG		MKKS	MKS1	MLC1
MLYCD	MMAA	MMAB	MMACHC		MMADHC		MME	MNF1	MOCOS	MOCS1	MOCS2	MOGS	MORC2	MPDU1	MPDZ	MPI	MPV17
MPZ	MR1	MRE11A	MRPL12	MRPL3	MRPL44	MRPS16	MRPS22	MRPS23	MRPS34	MRPS7	MRTO4	MSMO1	MSTO1	MSX2	MTCH1	MTERF	MTFMT
MTHFD1L		MTHFR	MTM1	MTMR14	MTMR2	MTO1	MTOR	MTPAP	MTR	MTRR	MUSK	MUT	MVK	MYBPC1	MYCN	MYF6	MYH14
MYH2	MYH3	MYH7	MYH8	MYO18B	MYO9A	MYOT	MYPN	MYT1L	NAA15	NACC1	NADK2	NAGA	NAGLU	NAGS	NALCN	NANS	NAPB
NARS2	NAT8L	NCAPD2	NCAPD3	NCAPH	NCKAP1	NDE1	NDP	NDRG1	NDST1	NDUFA1	NDUFA10		NDUFA11		NDUFA12		NDUFA13
NDUFA2	NDUFA3	NDUFA4	NDUFA4L2		NDUFA5	NDUFA6	NDUFA9	NDUFAF1		NDUFAF2		NDUFAF3		NDUFAF4		NDUFAF5	
NDUFAF6		NDUFB1	NDUFB10		NDUFB11		NDUFB2	NDUFB3	NDUFB4	NDUFB5	NDUFB6	NDUFB7	NDUFB8	NDUFB9	NDUFS1	NDUFS2	NDUFS3
NDUFS4	NDUFS5	NDUFS6	NDUFS7	NDUFS8	NDUFV1	NDUFV2	NDUFV3	NEB	NECAP1	NEDD4L	NEFH	NEFL	NEK1	NEK8	NEK9	NEU1	NEUROD1
NFIA	NFS1	NFU1	NGLY1	NGN3	NHEJ1	NHLRC1	NIN	NIPA1	NIPA2	NKX6-2	NLGN4X	NONO	NOTCH3	NPC1	NPC2	NPHP1	NPHP3
NPHP4	NPL	NPRL2	NPRL3	NRAS	NRXN1	NSDHL	NSUN2	NT5C2	NT5C3A	NTNG1	NTRK2	NUAK1	NUBPL	NUP37	NUS1	OAT	OCLN
OFD1	OGDH	OGT	OPA1	OPA3	OPHN1	ORAI1	OTC	OTUD6B	OTX2	OXCT1	P4HB	PABPN1	PACS1	PACS2	PAFAH1B1		PAH
PAK1	PAK3	PANK2	PARK2	PARK7	PARS2	PAX3	PAX4	PAX5	PBX1	PC	PCBD1	PCCA	PCCB	PCDH12	PCDH19	PCDH7	PCK1
PCK2	PCNA	PDE10A	PDE6D	PDGFB	PDHA1	PDHA2	PDHB	PDHX	PDK3	PDP1	PDP2	PDSS1	PDSS2	PDX1	PDYN	PEPD	PET100
PET117	PEX1	PEX10	PEX11B	PEX12	PEX13	PEX14	PEX16	PEX19	PEX2	PEX26	PEX3	PEX5	PEX6	PEX7	PFKFB2	PFKM	PGAM2
PGAP1	PGAP2	PGK1	PGM1	PHC1	PHF6	PHF8	PHIP	PHKA1	PHKA2	PHKB	PHKG2	PHYH	PIBF1	PIEZO2	PIGA	PIGC	PIGG
PIGH	PIGN	PIGO	PIGQ	PIGS	PIK3CA	PIK3R2	PIK3R5	PINK1	PIP5K1C	PKLR	PLA2G6	PLAA	PLCB1	PLD3	PLEC	PLEKHG2	
PLEKHG5		PLOD1	PLOD2	PLOD3	PLP1	PMM2	PMP22	PMPCA	PMPCB	PNKP	PNP	PNPLA2	PNPLA4	PNPLA6	PNPLA8	PNPO	PNPT1
POGLUT1		POGZ	POLG	POLG2	POLR1A	POLR1C	POLR2M	POLR3A	POLR3B	POLRMT	POMGNT1		POMGNT2		POMK	POMT1	POMT2
PPIB	PPM1D	PPOX	PPP1CB	PPP1R15B		PPP2R1A	PPP2R5D	PPP3CA	PPT1	PQBP1	PRDM8	PRICKLE1		PRICKLE2		PRKAG2	PRKCG
PRKRA	PRNP	PRODH	PROSC	PRPF39	PRPS1	PRRT2	PRSS12	PRUNE1	PRX	PSAP	PSMD12	PTCD1	PTCH1	PTCHD1	PTEN	PTPN11	PTS
PUM1	PURA	PUS1	PUS3	PYCR2	PYGL	PYGM	PYROXD1		QARS	QDPR	QRSL1	RAB11A	RAB11B	RAB18	RAB23	RAB33B	RAB39B
RAB3GAP1		RAB3GAP2		RAB40AL		RAB7A	RAC1	RAF1	RALGAPA1		RANBP2	RAPSN	RARS	RARS2	RBBP8	RBCK1	RBFOX1
RBFOX3	RBM12	RBMX	RECQL4	REEP1	REEP2	RELN	REPS1	RERE	RFT1	RHOBTB2		RIT1	RLIM	RMND1	RNASEH1		RNASEH2A
RNASEH2B		RNASEH2C		RNASET2		RNF170	RNF216	ROBO1	ROGDI	RORA	RORB	RP2	RPGRIP1L		RPIA	RPL10	RPS23
RPS6KA3	RRM2B	RTN2	RTN4IP1	RTN4R	RTTN	RYR1	RYR3	SACS	SAMD9L	SAMHD1	SARS	SARS2	SASS6	SBF1	SBF2	SC5D	SCA2
SCAPER	SCARB2	SCN10A	SCN1A	SCN1B	SCN2A	SCN3A	SCN4A	SCN7A	SCN8A	SCN9A	SCO1	SCO2	SCP2	SCYL1	SDCCAG8		SDHA
SDHAF1	SDHAF2	SDHB	SDHC	SDHD	SELENON		SEMA3E	SEPSECS	SERAC1	SERPINF1		SERPINH1		SERPINI1	SET	SETBP1	SETD2
SETD5	SETDB2	SETX	SFXN4	SGCA	SGCB	SGCD	SGCE	SGCG	SGSH	SH3TC2	SHANK2	SHANK3	SHH	SHOC2	SHROOM4		SIGMAR1
SIK1	SIL1	SIN3A	SIX3	SKI	SLC12A5	SLC12A6	SLC13A5	SLC16A1	SLC16A2	SLC17A5	SLC18A2	SLC18A3	SLC19A3	SLC1A1	SLC1A2	SLC1A3	SLC1A4
SLC22A5	SLC25A1	SLC25A12		SLC25A13		SLC25A15		SLC25A19		SLC25A20		SLC25A22		SLC25A26		SLC25A3	SLC25A32
SLC25A4	SLC25A42		SLC25A46		SLC2A1	SLC2A10	SLC2A2	SLC30A10		SLC30A9	SLC33A1	SLC35A1	SLC35A2	SLC35A3	SLC35C1	SLC37A4	SLC39A13
SLC39A14		SLC39A8	SLC3A1	SLC45A1	SLC46A1	SLC52A1	SLC52A2	SLC52A3	SLC5A1	SLC5A7	SLC6A1	SLC6A17	SLC6A19	SLC6A3	SLC6A5	SLC6A8	SLC6A9
SLC9A1	SLC9A6	SLC9A9	SMARCA1		SMARCA4		SMARCB1		SMARCC2		SMC1A	SMCHD1	SMPD1	SMS	SNAP25	SNAP29	SNORD118

SNX14	SOD1	SON	SOS1	SOS2	SOX10	SOX11	SOX2	SOX3	SP7	SPAST	SPATA5	SPEG	SPG11	SPG20	SPG21	SPG7	SPR
SPTAN1	SPTBN2	SPTBN4	SQSTM1	SRD5A3	SRGAP2	SRGAP2C		SRPX2	SSR4	ST3GAL3	ST3GAL5	STAC3	STAG1	STIL	STIM1	STRADA	STT3A
STT3B	STUB1	STX1B	STXBP1	STYXL1	SUCLA2	SUCLG1	SUCO	SUFU	SUMF1	SUOX	SURF1	SYN1	SYN2	SYNE1	SYNE2	SYNGAP1	
SYNJ1	SYP	SYT14	SYT1	SYT2	SZT2	TACO1	TAF1	TAF13	TAF2	TANGO2	TARDBP	TARS2	TAT	TAZ	TBC1D23	TBC1D24	TBC1D7
TBCD	TBCE	TBCK	TBL1XR1	TBR1	TCAP	TCF12	TCF2	TCF3	TCF4	TCF7L2	TCN2	TCTN1	TCTN2	TCTN3	TDGF1	TDP1	TDP2
TECPR2	TECR	TELO2	TENM4	TERF2	TFAM	TFG	TGFB1	TGFB2	TGFB3	TGFBR1	TGIF1	TGM6	TH	THAP1	THOC2	TIA1	TIMM50
TIMM8A	TIMMDC1		TK2	TKT	TLK2	TM4SF20	TMEM106B		TMEM107		TMEM126A		TMEM126B		TMEM138		TMEM165
TMEM199		TMEM216		TMEM231		TMEM237		TMEM240		TMEM38B		TMEM43	TMEM5	TMEM67	TMEM70	TMLHE	TMTC3
TNIK	TNNI2	TNNT1	TNNT3	TNPO3	TNXB	TOP3A	TOR1A	TOR1AIP1		TOR1AIP2		TPK1	TPM2	TPM3	TPP1	TRAF3IP1	
TRAIP	TRAK1	TRAPPC11		TRAPPC12		TRAPPC6B		TRAPPC9		TREX1	TRIM2	TRIM32	TRIM36	TRIM54	TRIM63	TRIM8	TRIO
TRIP12	TRIP4	TRIT1	TRMT10A		TRMT10C		TRMT5	TRMU	TRNT1	TROVE2	TRPC3	TRPV4	TSC1	TSC2	TSEN15	TSEN2	TSEN34
TSEN54	TSFM	TSPAN7	TTBK2	TTC19	TTC21B	TTC8	TTI2	TTN	TTPA	TTR	TUBA1A	TUBA8	TUBB	TUBB2A	TUBB2B	TUBB3	TUBB4A
TUBB6	TUBG1	TUBGCP4		TUBGCP6		TUFM	TUSC3	TWIST1	TXN2	TYMP	UBA1	UBA5	UBE2A	UBE3A	UBR3	UBR4	UBTF
UCHL1	UCP2	UFC1	UFM1	UMOD	UMPS	UNC13A	UNC80	UPB1	UPF3B	UQCC3	UQCR10	UQCR11	UQCRB	UQCRC1	UQCRC2	UQCRFS1	
UQCRH	UQCRHL	UQCRQ	UROC1	UROD	UROS	USP18	USP27X	USP8	USP9X	VAC14	VAMP1	VARS	VARS2	VCP	VDAC1	VDAC2	VLDLR
VMA21	VPS11	VPS13A	VPS13D	VPS33A	VPS37A	VPS53	VRK1	VWA3B	WAC	WARS	WARS2	WASHC4	WDFY3	WDR19	WDR26	WDR33	WDR45
WDR45B	WDR48	WDR62	WDR73	WDR81	WWOX	XDH	XRCC1	XRCC4	YARS	YARS2	YME1L1	YWHAG	YY1	ZAK	ZBTB18	ZBTB42	ZC3H14
ZDHHC15		ZDHHC9	ZEB2	ZFR	ZFYVE26	ZFYVE27	ZIC2	ZMYM2	ZMYM3	ZMYND11		ZNF148	ZNF335	ZNF41	ZNF423	ZNF469	ZNF592
ZNF674	ZNF711	ZNF81	ZSWIM6														
